# A Micromethod for Polyphenol High-Throughput Screening Saves 90 Percent Reagents and Sample Volume

**DOI:** 10.3390/antiox9010011

**Published:** 2019-12-21

**Authors:** Franz Tatzber, Willibald Wonisch, Sonja Lackner, Meinrad Lindschinger, Werner Pursch, Ulrike Resch, Christopher Trummer, Michael Murkovic, Sieglinde Zelzer, Sandra Holasek, Gerhard Cvirn

**Affiliations:** 1 Otto Loewi Research Center for Vascular Biology, Immunology and Inflammation, Devision of Immunology and Pathophysiology, Medical University of Graz, 8010 Graz, Austria; franz.tatzber@medunigraz.at (F.T.); sonja.lackner@medunigraz.at (S.L.); werner.pursch@aon.at (W.P.); sandra.holasek@medunigraz.at (S.H.); 2 Otto Loewi Research Center for Vascular Biology, Immunology and Inflammation, Devision of Physiological Medicine, Medical University of Graz, 8010 Graz, Austria; gerhard.cvirn@medunigraz.at; 3 Institute of Nutritional and Metabolic Diseases, Outpatient Clinic Laßnitzhöhe, 8301 Laßnitzhöhe, Austria; meinrad@lindschinger.at; 4 Department of Vascular Biology and Thrombosis Research, Medical University of Vienna, 1090 Vienna, Austria; uresch@gmx.at; 5 Institute of Biochemistry, Graz University of Technology, 8010 Graz, Austria; jobs@techsult.at (C.T.); michael.murkovic@tugraz.at (M.M.); 6 Clinical Institute of Medical and Chemical Laboratory Diagnostics, Medical University of Graz, 8010 Graz, Austria; sieglinde.zelzer@medunigraz.at

**Keywords:** antioxidants, Folin–Ciocalteu reagent, TAC, oxidative stress, TMB

## Abstract

There is ample evidence that polyphenols are important natural substances with pronounced antioxidative properties. This study aimed to develop a fast and reliable method to determine total polyphenol content (TPC) in foodstuffs and human samples. The microtitration format offers the advantage of low sample volumes in the microlitre range, facilitating high-throughput screening with 40 samples simultaneously. We accordingly adjusted the so-called Folin–Ciocalteu method to a microtitre format (polyphenols microtitre—PPm) with 90% reduction of reagents. The assay was standardized with gallic acid in the range between 0.1 and 3 mM, using a 20 µL sample volume. The intra-assay coefficient of variation (CV) was less than 5%, and inter-assay CV was in the range of 10%. Wavelength was measured at 766 nm after two hours of incubation. This micromethod correlates significantly with both the classical Folin–Ciocalteu method and High-Performance Thin-Layer Chromatography (HPTLC) (r^2^ = 0.9829). We further observed a significant correlation between PPm and total antioxidants (r^2^ = 0.918). The highest polyphenol concentrations were obtained for red, blue, and black fruits, vegetables, and juices. Extracts of red grapes could be harvested almost sugar free and might serve as a basis for polyphenol supplementation. Beer, flour, and bread contained polyphenol concentrations sufficient to meet the minimal daily requirement. We conclude that PPm is a sensitive and reliable method that detects polyphenols even in samples diluted 10-fold. The literature strongly recommends further investigations on the effects of polyphenol uptake on human and animal health.

## 1. Introduction:

There is overwhelming evidence that chain-breaking antioxidants have beneficial effects on free-radical-mediated disorders, such as atherosclerosis, acute myocardial infarction, and even a number of malignancies, which present the main causes of death in economically developed countries [1,2,3,4]. Polyphenols are important natural substances with pronounced antioxidative properties [5] that enable them to regulate oxidative stress in living organisms [6]. These phytochemicals possess a strong lipid-reducing activity [7], which promotes weight maintenance and provides a tool for an effective therapy for obesity and oxidative stress [8,9]. Considering that lipid peroxidation is a direct consequence of oxidative stress, adequate supplementation with antioxidants is desirable. Among substances with antioxidant properties, polyphenols also give very promising results beyond their main features as antioxidants, i.e., they provide anti-inflammatory [10], anti-atherogenic [11,12] and anticarcinogenic properties [13,14]. They are either taken up unmodified or become metabolised to substances with distinct antioxidant properties, which even depend on the cell type [15,16]. As polyphenols are secondary plant products with heterogeneous structures but a high potential for application in human health maintenance products, there is a certain demand for reliable, rapid, and specific methods for screening analysis of these substances from nutritional sources and from human serum and plasma samples. This seems particularly important in the context of a health claim that polyphenols in olive oil protect blood lipids against oxidative stress [17]. Consumers also demand more “natural” food additives, e.g., replacement of synthetic antioxidants with polyphenols to stabilize meat products [18]. These considerations led us to design a micromethod based on the well-established Folin–Ciocalteu assay [19,20], which allows simultaneous analysis of several samples at reasonable cost and application to several plant extracts and fruit juices, as well as to human samples. The method was applied to an acid test of efficacy, to determine the total polyphenol content (TPC) in different fruits, vegetables, juices, flour, bread, and beverages. The accuracy was compared with both the classical Folin–Ciocalteu assay and a high-performance thin-layer chromatography method (HPTLC). 

## 2. Materials and Methods

If not indicated otherwise, all reagents were obtained from Sigma-Aldrich (Vienna, Austria) or Honeywell Fluka–Fisher Scientific (Schwerte, Germany).

### 2.1. Microtitre Method for the Detection of TPC

Based on the method of Folin and Ciocalteu, we minimized sample and reagent volumes for microtitre plates. By this simple modification, we were able to reduce reagent volumes by 90%. The Folin–Ciocalteu reagent was diluted 1:10 before use, to avoid high coefficients of variation with respect to serial pipetting of small amounts. Briefly, a predilution of 1:10 was usually necessary, and was done on a predilution microtitration plate (Greiner, Kremsmünster, Austria), with 20 µL of sample diluted with 180 µL of distilled water. Then 20 µL of the diluted samples was applied to the assay plate. After sample application, 50 µL of distilled water was added to each well. Then 100 µL of the 1:10 prediluted Folin–Ciocalteu reagent was pipetted into each well. In a last step, 30 µL of Na_2_CO_3_ solution (0.7 mol in distilled water) was added to intensify the signal. Finally, the reaction was allowed to develop for at least 2 h at room temperature (wells were sealed with a foil), and the dark colour was measured at 766 nm. Serial dilutions of gallic acid were used for standardization, starting at 3 mM and using dilutions to give 1, 0.3, and 0.1 mM. Distilled water was used as blank. All analyses were carried out in duplicates. 

### 2.2. Folin–Ciocalteu Assay (FC-Assay)

#### 2.2.1. Sample Preparation 

To remove interfering substances in the FC Assay, solid-phase extraction Bond Elut C18 (200 mg, 6 mL, Agilent, Santa Clara, CA, USA) was performed prior to analysis. The stationary phase was conditioned, using 5 mL of methanol and 2 mL of distilled water. Thereafter, 4 mL of samples was applied to solid phase extraction, washed with 2 mL of distilled water and 2 mL of 20% methanol, to remove unbound substances. The sample was finally eluted with 1.5 mL of methanol. 

#### 2.2.2. Sample Dilution

Those samples used in the solid-phase extraction were diluted 1:3 with methanol. All other samples were diluted 1:10 with ethanol. 

#### 2.2.3. FC Assay Procedure

One milliliter of the extracted sample solution was pipetted into a volumetric flask, approximately 60 to 70 mL of distilled water was added, and the flask was swirled to mix the components. Then 5 mL of the Folin–Ciocalteu phenol reagent mixture (Sigma-Aldrich P/N F-9252, St. Louis, MO, USA) was added to the sample solution. After thorough mixing, between 1 and 8 min, 15 mL of sodium carbonate solution (20 g in 100 mL of water) was added. The solutions were mixed and diluted to 100 mL with distilled water, and again mixed by inverting the flask several times. The spectrophotometric measurement (using the Varian Cary 50 photometer) was performed after 2 h of incubation, at the maximum absorbance of 765 nm. 

#### 2.2.4. Preparation of Standards

A gallic acid stock solution was prepared (i.e., 1 mL of gallic acid solution (2 mg/mL) plus 1.5 mL of 40% ethanol). After cooling down, 40% ethanol was added to the mixture to give a final volume of 2.5 mL in the volumetric flask. A serial dilution with 40% ethanol was prepared: 1:2, 1:4, 1:8, 1:16, and 1:32.

### 2.3. HPTLC Method for TPC

#### 2.3.1. Thin-Layer Chromatography

The following equipment was used for thin-layer chromatography: CAMAG (Muttenz, Switzerland) Automatic TLC Sampler 4 (ATS 4); and double chamber (CAMAG). A densitometric slit scanner (CAMAG TLC Scanner) was used to visualise the chromatograms, and a Hetotrap CT 110 was used to cool them during their automated development. For chromatography, HPTLC plates (Silica gel 60 (Merck) 20 × 10 cm) were used, which were pretreated with methanol and heat-activated.

A 0.05 mM DPPH solution was prepared in MeOH, with an absorbance of ca. 0.4. Then 20 mL of this solution was poured into the separation chamber, to coat the plates with the detection reagent, and 10 µL of the samples was spotted onto the plates. The eluent was a mixture of toluene:ethyl-acetate:formic acid = 4:7:1 (v/v) for less-polar compounds, and ethyl acetate:water:formic acid = 17:2:2 (v/v) for medium and highly polar compounds. The following standard substances were used: catechin, gallic acid, gallotannins, caffeic acid, ascorbic acid, and naringin. 

For separation, a Twin Trough Chamber (CAMAG) was saturated for 20 min; the development distance was set to 80 mm, and the post-development drying time to 2 min. The plates were subsequently heated for 3 min at 100 °C, on a TLC Plate Heater III (CAMAG), and, after drying, they were instantly dipped in a 0.05 mM DPPH solution in methanol for 1 s, using Chromatogram Immersion Device III (CAMAG). The chromatograms were scanned before the DPPH reaction at 280 nm and after the DPPH reaction at 550 nm.

#### 2.3.2. Total Antioxidant Capacity

For the mesaurement of the total antioxidant capacity in respective samples, we used a commercially available colorimetric assay (TAC^®^, Labor Diagnostic Nord, Nordhorn, Germany). The method is based on the inhibition of radical mediated coloring of tetramethylbenzidine (TMB) by antioxidants [21,22]. Briefly, 25 µL of standards (Trolox: 0, 0.375, 0.75, 1.5, and 3 mmol) controls and samples were incubated with 100 µL of reagent A, consisting of 30% hydrogenperoxide and citrate-substrate buffer in a proportion of 1:1000 and 50 µL reagent B, consisting of horseradish peroxidase (25 mU), TMB, and a citrate-substrate buffer in a proportion of 1:10:1000, in uncoated microtitre plates. After an incubation period of 20 min at 4 °C in the dark, the reaction was stopped by the addition of the acidic stop solution (50 µL of 2N sulfuric acid), and absorbance reading was done at a wavelength of 450 nm (reference 620 nm), in a microplate photometer. 

#### 2.3.3. Sample Preparation

##### Fruits and Vegetables

Fruit and vegetable samples were treated according to their water content. If the water content was >30% of fresh weight, fruits and vegetables were homogenized with a homogenizer. The homogenates were centrifuged for 15 min at 2000× *g*, in a laboratory centrifuge (Heraeus), and the clear supernatant was used for analysis. With insufficient phase separation, turbid supernatants were centrifuged at 8000× *g* for 4 min, in a high-speed centrifuge (Eppendorf, Hamburg, Germany), and the clear supernatants were analysed for polyphenols.

Fruits and vegetables with low water content (<30%) were weighed, and distilled water was added to the weight (mL/g) before homogenization, e.g., if a sample weighed 5.6 g, we added 5.6 mL of distilled water. For dry samples (e.g., flour), we added the 10-fold amount of water. The remaining procedure was as described before.

##### Beer Samples

Different beer samples (10 mL) were collected and stored at 4 °C, until use. Clear samples were allowed to reach room temperature and analysed for polyphenols from 1:10 predilutions. Turbid samples were centrifuged at 8000× *g* in a high-speed centrifuge, and the clear supernatants were used for analysis.

##### Coffee, Instant Coffee, and Tea

Coffee prepared from roasted coffee beans in a fully automated coffee machine (deLonghi, Treviso, Italy) was analysed for polyphenols.

Instant coffee was weighed, and the 10-fold amount of distilled water was added. After centrifugation, the clear supernatant was analysed.

Tea was weighed and brewed with 50 mL of boiling distilled water. Aliquots were taken after 3, 6, 12, 25, and 50 min, to analyse polyphenols and antioxidants. All samples were allowed to reach room temperature before analysis.

##### Flour and Bread

Flour and bread were weighed, and the 10-fold amount of 50% ethanol was added to each sample (mL/g). Polyphenols were extracted for 24 h on a rocking device (Heidolph, Schwabach, Germany), at 1 revolution sec^−1^. After incubation, the samples were centrifuged as described above, and the clear supernatants were analysed.

##### Extraction of Polyphenols from Red Grapes

Residues from black grapes after pressing were dried at 60 °C for 48 h and homogenized with a grinder. Dry matter was weighed, and 50% ethanol was added in the ten-fold volume of the sample weight (mL/g). For extraction, the grape powder was incubated in ethanol for 24, 48, 72, 96, 120, and 144 h. After incubation, the samples were treated as described before. 

#### 2.3.4. Statistics

The SigmaPlot package 10.0 (SPSS, Erkrath, Germany) was used for statistical analysis. Groups (light vs. strong beers) were compared with the t-test. Correlations between methods were performed with the Spearman´s rank-order correlation. Results were expressed as mean ± standard deviation (SD), and *p* < 0.05 was considered significant.

## 3. Results

### 3.1. Evaluation of the Microplate Polyphenol Assay

**Figure 1:**
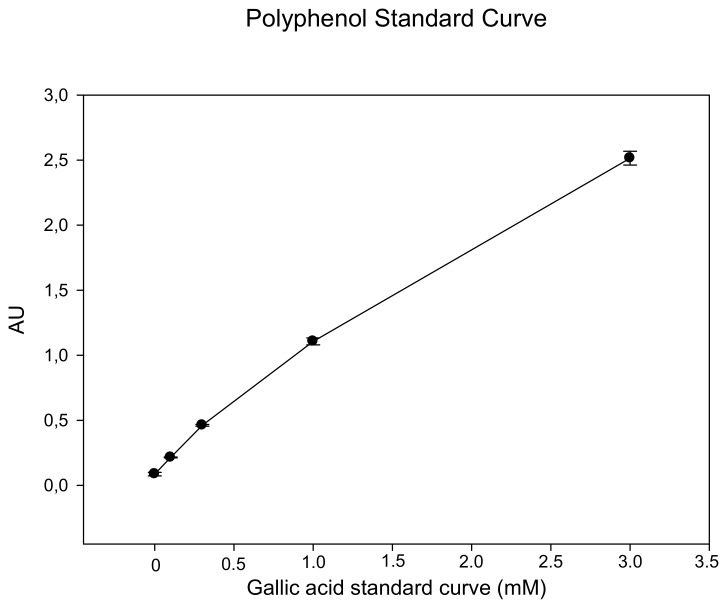
Gallic acid standard curve (mM). The range between 0.3 and 3 mM is linear. Samples with low polyphenol concentrations (<0.3 mM) should be applied undiluted.

Absorption signals ranged from <0.1 Absorption Units (AU) in blanks to 2.5–3 AU in the 3 mM standard. The intra-assay coefficient of variation (CV) was found to be <5% (number of replicates ×10). Inter-assay CV was in the range of 10%. A control sample of 0.6–1 mM was found to give an inter-assay CV of 11.6% (*n* = 47 assays). Limit of detection (LOD) and limit of quantification (LOQ) were calculated to be 30 and 99 µM, respectively. Figure 1 shows a typical standard curve. Samples with polyphenol concentrations <2 mM should be used undiluted in the assay, because results obtained from 1:10 prediluted samples showed a certain tendency to overestimate polyphenol concentrations.

### 3.2. PPm in Comparison with FC Assay and HPTLC

**Figure 2:**
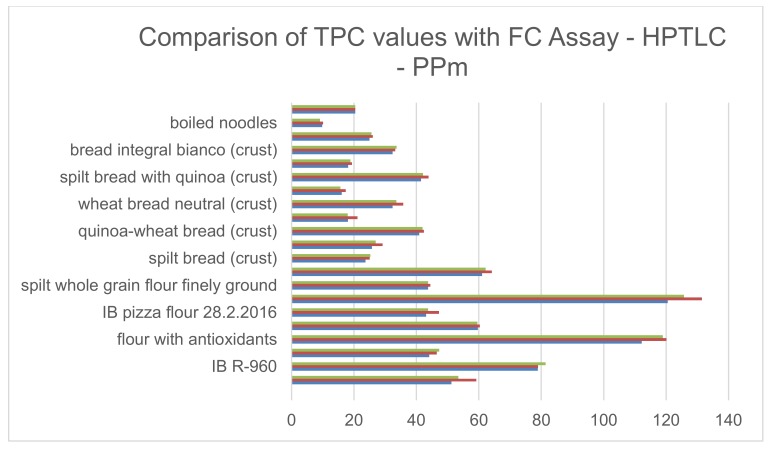
Comparison of TPC with the Folin–Ciocalteou (FC) Assay by the use of a Cuvette (green line)—HPTLC (red line) and PPm (blue line) in diverse flours, breads (crumb and crust), and noodles. These methods correlated significantly with a correlation coefficient of (r^2^ = 0.9829). Values are presented as mg/mL.

TPC was measured in diverse flours, breads (crumb and crust), and noodles, with three different methods, i.e., micromethod (PPm), classical FC assay, and HPTLC. These methods correlated significantly with a correlation coefficient of (r^2^ = 0.9829). For details, see Figure 2 [23].

### 3.3. PPm and Total Antioxidant Capacity

**Figure 3:**
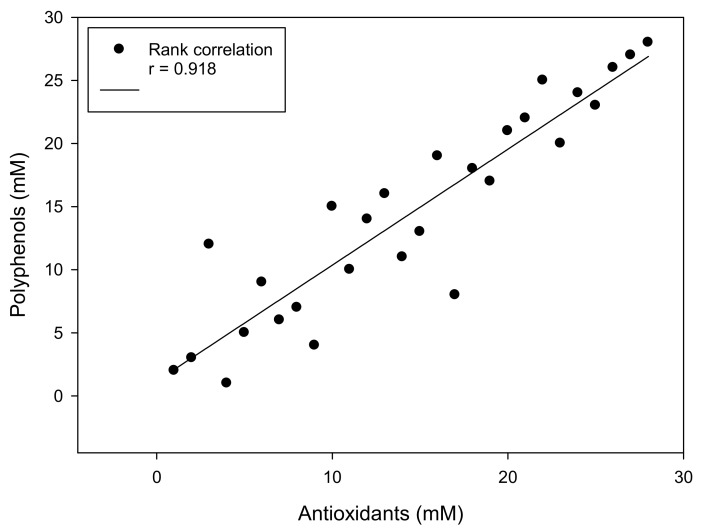
Rank correlation of antioxidants (*x* axis) and polyphenols (*y* axis). All data are expressed as mM.

Total polyphenol content and the total antioxidant capacity correlated significantly with a rank-correlation coefficient of (r^2^ = 0.918; *p* < 0.0001) (see Figure 3).

### 3.4. Polyphenol Content in Fruits and Vegetables

Results obtained for fruits and vegetables are summarized in Table 1. From the selected series, black currants, elderberries, lovage, and peppermint showed the highest concentrations of polyphenols, while cucumbers and zucchini contained the lowest. With extraction from herbs, polyphenol concentrations of water extracts were higher than those in ethanol extracts. This finding contradicts the results obtained for grapes, where higher polyphenol concentrations were found in ethanol extracts.

### 3.5. Polyphenol Content in Beer

Highly significant correlations in Spearman´s rank correlation were found between polyphenol concentrations and original gravity of the beers (*n* = 130, r^2^ = 0.378, *p* < 0.01). Ethanol concentrations and polyphenol content correlated significantly as well (r^2^ = 0.359, *p* < 0.01). As a consequence, significant differences were found between light and strong beers in the t-test (*p* < 0.01). In contrast, no significant differences were found for beer stored in bottles compared to barrel storage. Polyphenol concentrations in beer samples gave concentrations from 2 to 9 mM, which is comparable to the polyphenol content, e.g., in apples. A more detailed analysis of the samples revealed that lager beers gave better polyphenol concentrations than pils types (*t*-test, *p* < 0.05). It could also be shown that beer contained remarkable concentrations of antioxidants in the millimolar range. 

### 3.6. Polyphenol Content in Coffee, Instant Coffee, and Tea

In 28 tea samples of various origins (herbal tea and flavoured and unflavoured Russian tea), we found a clear correlation between total antioxidants and polyphenol content. As expected, total antioxidants gave somewhat higher concentrations than polyphenols. 

Polyphenol concentrations in different tea varieties ranged from 5.38 (ginger–lemon tea) to 27.3 mM (jasmine tea). Coffee samples of 1% (w/v) were found at 3–5 mM.

### 3.7. Polyphenol Concentrations in Flour and Bread

**Figure 4:**
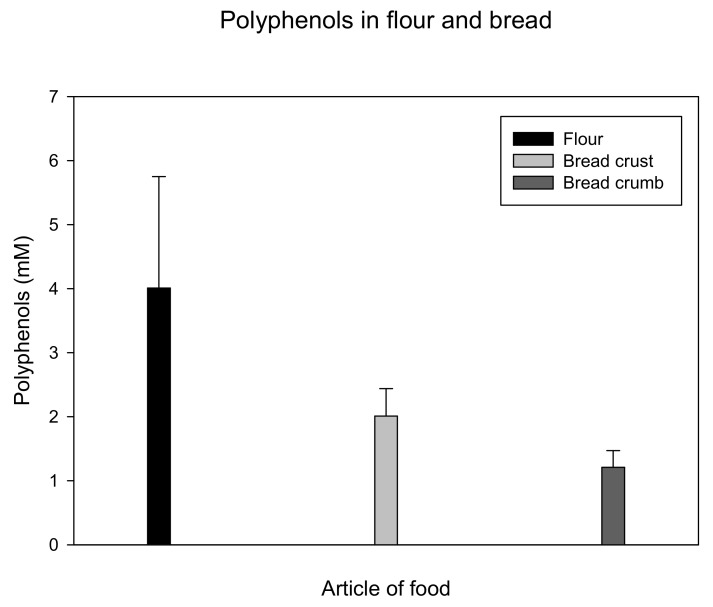
Polyphenols (mean ± standard deviation) of flour and bread. Approximately half of the polyphenol content is lost during the baking process.

In flour samples, polyphenol concentrations were in the range of 3 mM and doubled if enriched with antioxidants. However, after baking, only half of the polyphenol concentrations were found in bread crust, and even less were found in bread crumbs. Details are presented in Figure 4.

### 3.8. Polyphenol Extracts from Red Grapes

**Figure 5:**
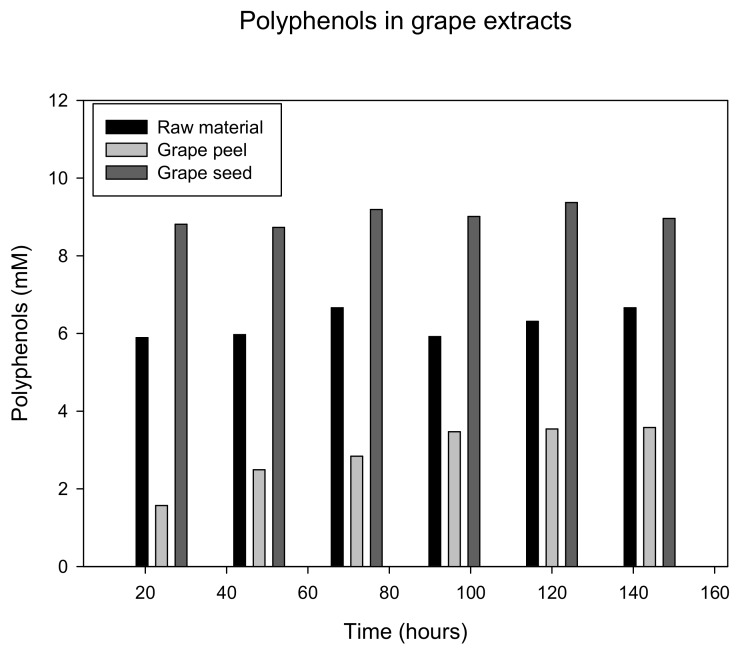
Results of extraction of red grapes with 50% ethanol. Samples were determined in duplicate after 24, 48, 72, 96, 120, and 144 h and are expressed as means.

Best extraction results were obtained with 50% ethanol. In addition to the starting material, grape samples were divided into seeds and peels by sieving. These experiments showed that, by far, the highest polyphenol concentrations could be extracted from seeds almost instantly. In contrast, extraction of polyphenols from grape skins was time-dependent and yielded much lower concentrations. These data are presented in Figure 5. 

## 4. Discussion

Based on a microtitre modification of the Folin–Ciocalteu method for detection of polyphenols, we investigated several possible sources of polyphenol in food. With this microtitre modification, neither sample numbers nor sample amounts are significant limiting factors, because only microlitre sample volumes are necessary, and a sample size of 40 specimens can be measured simultaneously on one microtitration plate, in duplicates. Standardisation was done with gallic acid, in the range between 0.1 and 3 mmol/L. Results were expressed as gallic acid equivalents in mmol/L (GAE mmol/L), because we found similar results in equimolar amounts of polyphenols with this assay modification. Colouring of samples did not interfere with the colour of the reagent, because most samples were prediluted at least 1:10 before assaying, and spectra from extracts did not show absorptions at 766 nm. Intra- and inter-assay coefficients of variation were found in the range of 5% and 10%, respectively. Stability experiments showed no changes in properties of reagents or standards for at least six months after production, if they were refrigerated. The high correlation between total polyphenols and total antioxidants with a correlation coefficient of (r^2^ = 0.918) is consistent with a previous report of Namiesnik et al. [24] and provides an additional advantage, because both methods measure in the same determination range, which simplifies sample preparation significantly. 

From our findings, it can be stated that most fruits, herbs, and vegetables, and of course their juices, are valuable sources of polyphenols. Blackcurrant was among those fruits with highest TPC, which is consistent with Abountiolas and Nunes [25], who further observed high stability for the polyphenols in blackcurrant juice during refrigerated storage. Better stability was found at a storage temperature of 4–15 °C than at 25 °C [26,27]. A combination of different polyphenol sources counteracts degradation as well [28]. Peels and skins usually contain higher concentrations of polyphenols than pulp. Beer, tea, and coffee also contained remarkable amounts of polyphenols and therefore may be used as polyphenol sources as well. This is in accordance with a consensus document indicating that moderate beer consumption reduces the risk of cardiovascular disease [29]. Polyphenols are thermally unstable, because flour and bread lose about 50% of their initial polyphenols during the baking process. A pronounced decrease of TPC as high as 90 percent was reported for jam and marmalade processing [27]. 

Disorders like diabetes or celiac disease might lead to lack of polyphenols and insufficient antioxidative capacities of those patients. With this in mind, we tested whether, besides tea and coffee, press residues of red grapes might serve as sources of polyphenol extracts. The skin of red grapes is of particular interest due to its high resveratrol content, which is responsible for both anticancer activities, including cell-cycle effects and anti-inflammatory effects [30]. In an animal experiment, wine lees were shown to be a rich source of polyphenols as a basis for functional food without alcohol. They were able to compensate for an unhealthy lifestyle in the case of a high-cholesterol diet, reducing oxidative stress levels and antioxidant enzymes to control values [31]. Results of these experiments are in agreement with a previous report [32] that emphasized the seeds of red grapes as being good sources for polyphenols. It might be assumed that, with adequate nutrition, polyphenols will provide a sufficient level for antioxidative protection, though diabetic and celiac patients could require polyphenol and antioxidant supplementation, perhaps as red grape extracts without sugar and flour. Due to the adaptations in the PPm assay, this method is also suitable for human specimens, as has been shown previously [21] with respect to work intensity between office workers and manual laborers. In a previous clinical pilot study (Lindschinger et al., accepted in MMW; Springer Verlag), we observed an unexpected decrease of the total polpyphenols in serum of the study participants at the end of supplementation (after six weeks) with a vitamin B complex in the range of 2.5 RDA, in spite of a significant increase of each B vitamin. Due to the crossover design of this study, we were able to show that this effect was reproducible with both natural and synthetic B vitamins. This suggests consumption of polyphenols to regenerate or protection of B vitamins, and it predestines the PPm as a tool to monitor the in vivo bioavailability of phytochemicals, e.g., in therapy monitoring. 

## 5. Conclusions

It can be concluded that this microtitre modification of the Folin–Ciocalteu method facilitates the analysis of a large number of samples with minimal sample volumes, which was acknowledged by the Austrian patent office as a utility model. Time-saving with the determination of 40 samples simultaneously is an additional benefit of this innovation. The foodstuff industry might take advantage of rapid polyphenol determination with this high-throughput method. This ensures safety for consumers with respect to an excess of antioxidants that might cause a pro-oxidative state. The possible application in human samples provides a large spectrum ranging from scientific issues via discovering new biochemical interactions through healthcare. Last but not least, this microtitre method is eco-friendly, stands out due to a convenient handling, and is cost-effective. Our findings are in accordance with results from other research groups. Further studies are necessary to evaluate bioavailability, antioxidant capacity, and health benefits in humans after polyphenol supplementation with foods and beverages containing polyphenol such as chokeberry juice [33]. 

## Figures and Tables

**Table 1:** Polyphenol concentrations of different herbs, vegetables, and fruits.

0–1.99 mM	2–3.99 mM	4–7.99 mM	8–15.99 mM	>16 mM
Cucumber	Peach	Paprika yellow	Paprika red	Currant black
Zucchini	Apricot	Grape white	Blackberry	Peppermint
	Tomato	Plum	Currant red	Lovage
	Apple	Apple		Grape red
	Asparagus	Parsley		Elderberry
	Walnut	Chive		
